# Downregulation of hsa-miR-132 and hsa-miR-129: non-coding RNA molecular signatures of Alzheimer’s disease

**DOI:** 10.3389/fnmol.2024.1423340

**Published:** 2024-06-25

**Authors:** Siranjeevi Nagaraj, Carolina Quintanilla-Sánchez, Kunie Ando, Lidia Lopez-Gutierrez, Emilie Doeraene, Andreea-Claudia Kosa, Emmanuel Aydin, Jean-Pierre Brion, Karelle Leroy

**Affiliations:** Alzheimer and Other Tauopathies Research Group, Faculty of Medicine, ULB Center for Diabetes Research, ULB Neuroscience Institute, Université Libre de Bruxelles, Brussels, Belgium

**Keywords:** Alzheimer’s disease (AD), microRNA, miR-132, miR-129, therapeutics

## Abstract

Alzheimer’s disease (AD) affects the elderly population by causing memory impairments, cognitive and behavioral abnormalities. Currently, no curative treatments exist, emphasizing the need to explore therapeutic options that modify the progression of the disease. MicroRNAs (miRNAs), as non-coding RNAs, demonstrate multifaceted targeting potential and are known to be dysregulated in AD pathology. This mini review focuses on two promising miRNAs, hsa-miR-132 and hsa-miR-129, which consistently exhibit differential regulation in AD. By employing computational predictions and referencing published RNA sequencing dataset, we elucidate the intricate miRNA-mRNA target relationships associated with hsa-miR-132 and hsa-miR-129. Our review consistently identifies the downregulation of hsa-miR-132 and hsa-miR-129 in AD brains as a non-coding RNA molecular signature across studies conducted over the past 15 years in AD research.

## Introduction

Alzheimer’s disease (AD) is the leading cause of dementia, presenting a significant socioeconomic burden within aging societies. AD is a progressive disorder that manifests at the molecular level long before clinically observable dementia symptoms emerge (Aisen et al., 2017; Knopman et al., 2021). It commences with a preclinical phase, characterized by the absence of symptoms, followed by mild cognitive impairment (MCI), progressing through mild, moderate, and severe late AD stages (Aisen et al., 2017; Jack et al., 2018). In over 95 percent of the cases, AD develops later in life (above 65 years) and does not directly result from genetic inheritance (Andrieu et al., 2015). Neuropathologically, AD is identified by the presence of extracellular amyloid plaques primarily composed of amyloid beta (Aβ) peptides and intracellular neurofibrillary tangles (NFTs) consisting of hyperphosphorylated tau (p-tau) in the hippocampus and cortex (Glenner and Wong, 1984; Brion et al., 1991; Buée et al., 2000; Congdon and Sigurdsson, 2018; Panza et al., 2019). The complex nature of the disease has posed challenges in pinpointing its exact cause, leading to a lack of curative treatments. Consequently, there is an urgent need to explore neuroprotective strategies that can modify the disease progression during its early stages. Noncoding RNAs are known for their regulatory roles in fine tuning the transcription and translation processes (Szafranski et al., 2015; Idda et al., 2018). MicroRNAs (miRNAs) are a subclass of short RNA molecules that regulate gene expression following transcription. On average they have 22 nucleotides, and they are expressed ubiquitously. They bind to specific regions of target genes [primarily at the 3′ untranslated region (UTR), though not limited to it], resulting in reduced expression of target genes through degradation of mRNA or inhibition of translation (Bartel, 2018; Nagaraj et al., 2021). Emerging evidence suggests that miRNAs undergo dysregulation in the brain, cerebrospinal fluid (CSF), and blood of individuals with AD (Nagaraj et al., 2019; Takousis et al., 2019; Yoon et al., 2022).

## Consistent downregulation of hsa-miR-132 and hsa-miR-129 in Alzheimer’s disease brain

**FIGURE 1:**
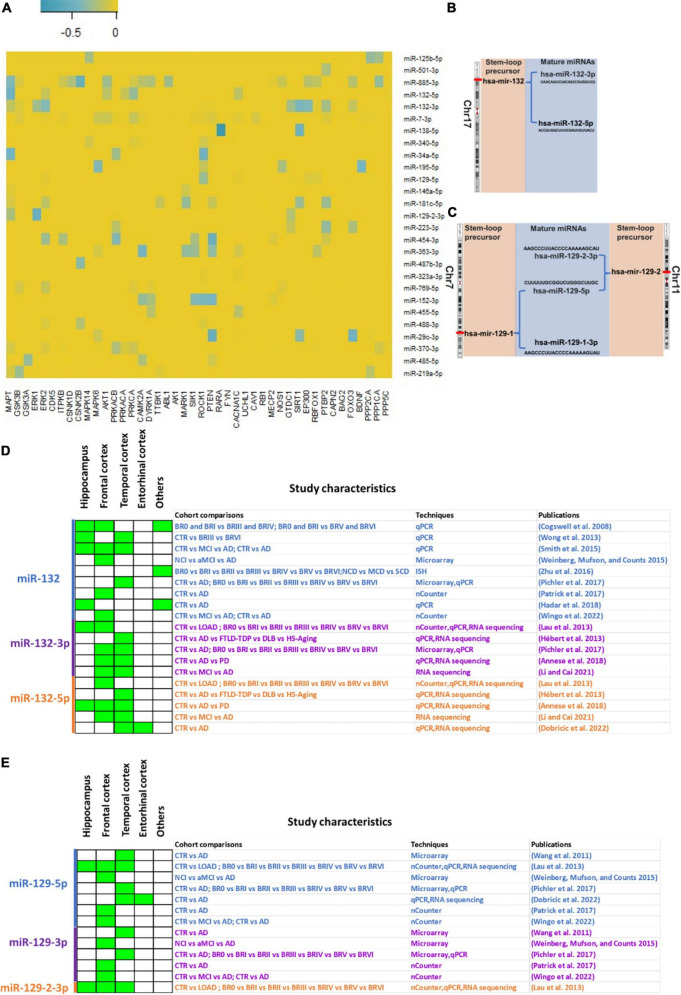
miRNA-mRNA target predictions and expression of hsa-miR-132 and hsa-miR-129 in *post-mortem* AD brain tissue. **(A)** On *Y*-axis of the heatmap, most promising dysregulated miRNAs in AD brain are shown (Patrick et al., 2017; Nagaraj et al., 2019; Takousis et al., 2019) and on *X*-axis of the heatmap, mRNA targets involved in direct and associated pathways in tau pathology. Blue gradient is used to visualize the prediction strengths based on TargetScan context score. Darker blue indicates the stronger predictions (most negative TargetScan context score) and light blue indicates the weaker predictions (least negative TargetScan context score) **(B,C)** Chromosomal location of hsa-miR-132 **(B)** and hsa-miR-129 (hsa-miR-129-1 and hsa-miR-129-2) **(C)** with the subsequent process to obtain mature miRNAs. **(D,E)** hsa-miR-132 **(D)** and hsa-miR-129 **(E)** differential expression level in various brain regions across AD studies. Green for downregulation in AD brains compared to control brains. Study characteristics show the cohort comparisons and techniques used in respective studies. “miR-” indicated in the figure refer to “hsa-miR.”

Several miRNAs are dysregulated in the brains of AD patients across various brain regions compared to non-demented controls (Nagaraj et al., 2019; Takousis et al., 2019). Most promising miRNAs dysregulated in AD brains and their predictions to bind mRNA targets involved in tau pathology are shown in Figure 1A. Of note, only few of these predictions are experimentally validated and demands further deciphering. Interestingly, recent studies validated hsa-miR-132 and hsa-miR-129 as standout miRNAs in terms of downregulation in AD patients (Patrick et al., 2017; Wingo et al., 2022). In this mini review article, we focus primarily on two miRNAs (hsa-miR-132 and hsa-miR-129) and discuss about their constant downregulation across different brain regions in AD, according to diverse cohorts (Figures 1B–E). We shed lights on their location, expression level and miRNA-mRNA target relationship in the *post-mortem* brains.

The gene for hsa-miR-132 is located on chromosome 17 in the p arm (17p13.3). The transcribed hsa-miR-132 stem loop hairpin precursor is then processed to generate two mature miRNAs: hsa-miR-132-3p (UAACAGUCUACAGCCAUGGUCG) and hsa-miR-132-5p (ACCGUGGCUUUCGAUUGUUACU) (Figure 1B). On the other hand, hsa-miR-129-5p is a mature miRNA derived from two possible stem loop hairpin precursors, each located on different chromosomes. The first precursor, hsa-miR-129-1, is situated on chromosome 7 in the q arm (7q32.1). After processing, it produces two mature miRNAs: hsa-miR-129-5p (CUUUUUGCGGUCUGGGCUUGC) and hsa-miR-129-1-3p (AAGCCCUUACCCCAAAAAGUAU). The second precursor, hsa-miR-129-2, is located on chromosome 11 in the p arm (11p11.2). Upon processing, it generates two mature miRNAs: hsa-miR-129-5p (CUUUUUGCGGUCUGGGCUUGC) and hsa-miR-129-2-3p (AAGCCCUUACCCCAAAAAGCAU) (Figure 1C).

There is consensus about the downregulation of hsa-miR-132 in AD across several studies (Figure 1D). Those focusing on the hippocampus, an early affected region, have yielded valuable insights into the differential expression level of miRNAs in this brain region. Cogswell et al. (2008) conducted qPCR experiments, revealing downregulation of hsa-miR-132 in the hippocampus, the frontal cortex and the cerebellum. Similarly, several studies have investigated miRNA expression with different methodologies in both hippocampal and cortical regions of the brain. For instance, Lau et al. (2013) employed nCounter, qPCR, and RNA sequencing techniques, detecting downregulation of hsa-miR-132 not only in the hippocampus but also in the prefrontal cortex and the temporal cortex. Likewise, Smith et al. (2015) performed qPCR and observed downregulation of hsa-miR-132 in the hippocampus, the frontal cortex, and the temporal cortex. Also, Annese et al. (2018) used qPCR and RNA sequencing, revealing downregulation of hsa-miR-132-5p and hsa-miR-132-3p not only in the hippocampus but also in the temporal gyrus and the frontal gyrus. In a different cohort, Hadar et al. (2018) performed qPCR experiments and found downregulation of hsa-miR-132 in the hippocampus.

Due to limitation in *post-mortem* hippocampal tissue availability, most of the studies focused solely on the cortex to identify the change in expression of miRNAs along the course of disease development. For example, Hébert et al. (2013) used qPCR and RNA sequencing, identifying downregulation of hsa-miR-132-5p in the temporal cortex. Wong et al. (2013) also found downregulation of hsa-miR-132 using qPCR in the temporal cortex. Pichler et al. (2017) utilized microarray and qPCR, detecting downregulation of hsa-miR-132 in the frontal cortex and temporal cortex. Additionally, Patrick et al. (2017) employed nCounter and found downregulation of hsa-miR-132 in the prefrontal cortex. Li and Cai (2021) used RNA sequencing, detecting downregulation of hsa-miR-132-5p and hsa-miR-132-3p in the inferior frontal gyrus and superior temporal gyrus (Li and Cai, 2021). Similarly, Dobricic et al. (2022b) used qPCR and RNA sequencing to identify the downregulation of hsa-miR-132-5p in the superior temporal gyrus and entorhinal cortex. In addition to hippocampus and cortex, other regions have been explored. For instance, Zhu et al. (2016) conducted *in situ* hybridization (ISH) experiments, identifying downregulation of hsa-miR-132 in the Nucleus basalis of Meynert. In a separate study by Hadar et al. (2018) using qPCR demonstrated significant downregulation of hsa-miR-132 in the olfactory bulb.

hsa-miR-129 is another miRNA that is consistently downregulated in AD (Figure 1E). Wang et al. (2011) observed downregulation of hsa-miR-129-3p and hsa-miR-129-5p in the superior and middle temporal gyri using microarray. In another study, Lau et al. (2013) reported downregulation of hsa-miR-129-5p and hsa-miR-129-2-3p in the hippocampus and in the cortex using nCounter, qPCR, and RNA sequencing techniques. A study from Weinberg, Mufson, and Counts used microarray and detected downregulation of hsa-miR-129* (hsa-miR-129-5p) and hsa-miR-129-3p in the frontal cortex (Weinberg et al., 2015). Pichler et al. (2017) performed microarray, identifying downregulation of hsa-miR-129-3p and hsa-miR-129-5p in the temporal cortex. In another study, Patrick et al. (2017) and Wingo et al. (2022) utilized nCounter, finding downregulation of hsa-miR-129-5p and hsa-miR-129-3p in the prefrontal cortex. Additionally, Dobricic et al. (2022b) reported downregulation of hsa-miR-129-5p in the superior temporal gyrus and entorhinal cortex through qPCR and RNA sequencing.

## Complexity in miRNA and mRNA target relationship– focus on hsa-miR-132 and hsa-miR-129

**FIGURE 2:**
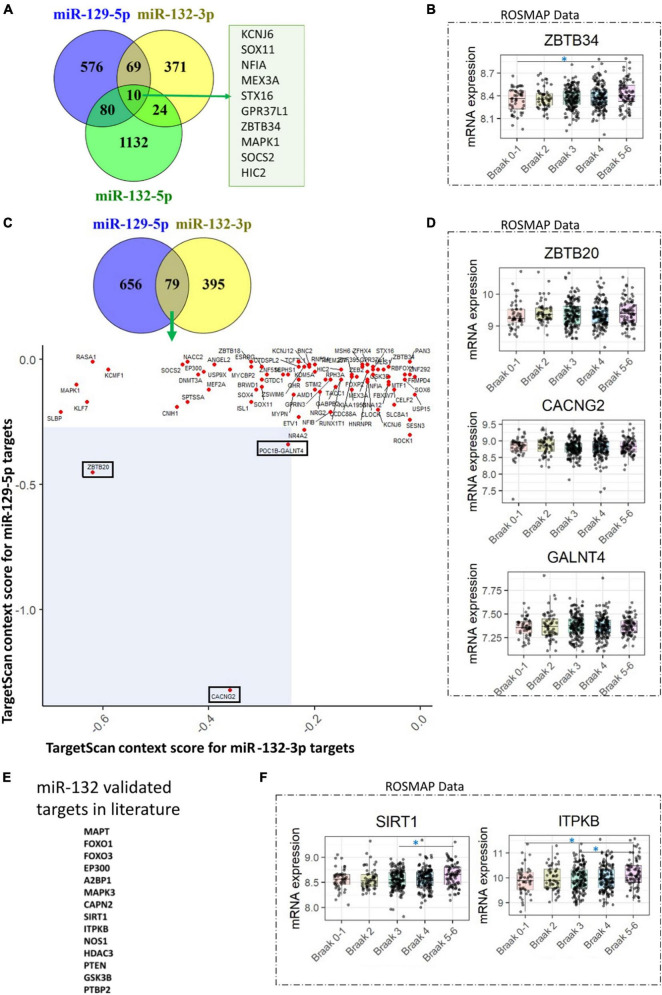
hsa-miR-132 and hsa-miR-129 target predictions and evaluation of mRNA levels in the ROSMAP dataset. **(A)** Venn diagram illustrating the targets identified for hsa-miR-132-3p, hsa-miR-132-5p, and hsa-miR-129-5p in the TargetScan database. A total of 10 targets common to all three miRNAs are shown. **(B)** Among the 10 targets identified in **(A)**, only one (ZBTB34) exhibited changes across the Braak stages in the ROSMAP dataset. **(C)** Venn diagram depicting the targets identified for hsa-miR-129-5p and hsa-miR-132-3p in the TargetScan database. The scatter plot shows 79 targets common to these two miRNAs, based on the TargetScan context score. Three selected targets, ZBTB20, CACNG2, and GALNT4, are highlighted with rectangles in the blue shaded area with total context score less than –0.25. **(D)** None of the three selected targets mentioned in **(C)** demonstrated changes across the Braak stages in the ROSMAP dataset. **(E)** miR-132 validated targets found in the literature. **(F)** Two targets, SIRT1 and ITPKB1, identified in **(E)**, exhibited changes across the Braak stages in the ROSMAP dataset. A one-way ANOVA with Tukey’s *post hoc* analysis was utilized. The symbol “*” indicates a *p*-value of less than 0.05. “miR-” indicated in the figure refer to “hsa-miR.”

Based on the aforementioned evidence, we have concluded that there is widespread downregulation of hsa-miR-132 and hsa-miR-129 in AD. As a result, we aimed to predict the targets of hsa-miR-132-3p, hsa-miR-132-5p, and hsa-miR-129-5p from TargetScan database^1^ (Agarwal et al., 2015). Subsequently, we assessed the levels of these predicted mRNA targets in dorsolateral prefrontal cortex using data from the ROSMAP cohort dataset (Jager et al., 2018).^2^ For hsa-miR-132-3p, hsa-miR-132-5p, and hsa-miR-129-5p, a total of 10 common targets were identified: KCNJ6, SOX11, NFIA, MEX3A, STX16, GPR37L1, ZBTB34, MAPK1, SOCS2, and HIC2 (Figure 2A). Upon analyzing the differences in their levels across Braak stage comparisons, only the mRNA of ZBTB34 exhibited increased levels in Braak 5-6 compared to Braak 0-1 (Figure 2B). Of note, in an independent study, increase of ZBTB34 mRNA levels have been showed in AD cortex compared to controls (Salta et al., 2016). Further, based on published studies we found that in contrast to hsa-miR-132-3p, the expression of hsa-miR-132-5p was found to be lower in AD brains (7.5 times) and in frontotemporal lobar degeneration with TDP-43 inclusions (FTLD-TDP) brains (50 to 100 times) (Chen-Plotkin et al., 2012; Lau et al., 2013). Therefore, we focused on investigating the common targets of hsa-miR-132-3p and hsa-miR-129-5p, that are abundantly expressed. Out of 79 identified targets, three targets (CACNG2, ZBTB20, and GALNT4) that displayed most negative total context score in TargetScan (less than cut off value of −0.25 for both hsa-miR-132-3p and hsa-miR-129-5p targets) (Figure 2C). However, none of these three mRNA targets showed increased levels as the disease progressed (based on Braak stages) (Figure 2D). Alternatively, numerous studies have validated various targets for miR-132 in AD literature (Salta and Strooper, 2017; Fatimy et al., 2018; Zhang and Bian, 2021). So, we wondered about the validated targets of miR-132 (such as MAPT, FOXO1, FOXO3, EP300, A2BP1, MAPK3, CAPN2, SIRT1, ITPKB, NOS1, HDAC3, PTEN, GSK3B, PTBP2) (Figure 2E) and how they might change in the ROSMAP dataset. After examining this, we found that SIRT1 and ITPKB levels increased as the disease progressed (based on Braak stages) (Figure 2F). Interestingly, while ITPKB protein levels demonstrated a corresponding increase in the AD cortex and hippocampus (Stygelbout et al., 2014; Salta et al., 2016), reports suggest a contrasting decrease in SIRT1 protein levels in later stages of AD (Julien et al., 2009). The role of ZBTB34 in AD is not clearly understood; however, the function of ITPKB as a regulator of extracellular signal-regulated kinases 1/2 activation is well established (Stygelbout et al., 2014). Additionally, the role of SIRT1 as a deacetylase, known for deacetylating tau protein at multiple residues, is documented (Min et al., 2018).

## Conclusion

Overall, these studies consistently demonstrate, through different methodologies, the downregulation of hsa-miR-132 and hsa-miR-129 in various regions of the AD brain, including the hippocampus, the cortex, and the olfactory bulb. While the downregulation of hsa-miR-132-3p and hsa-miR-129-5p is notably observed in AD, similar downregulation patterns are also identified in the brains of other neurodegenerative conditions, including Parkinson’s disease (Dobricic et al., 2022a). The regulatory effects of these miRNAs in both pathologies are yet to be fully understood. Furthermore, our observations indicate that only a few of the predicted targets exhibit an inverse relationship with the corresponding miRNA, while most of them remain unchanged. This intricate miRNA-mRNA interactions require further investigation to comprehend the mechanisms underlying AD. Additionally, factors such as the RNA integrity number (RIN) value may introduce bias into the findings. Moreover, a comprehensive understanding of proteomics data is highly necessary to properly identify the relevant core hub for these hsa-miR-132 and hsa-miR-129 targets in AD. Furthermore, most of the literature identifying dysregulated miRNAs in human AD post-mortem samples has used bulk sequencing techniques (Lau et al., 2013; Patrick et al., 2017; Dobricic et al., 2022a). This type of sequencing does not provide information about cell-type-specific miRNAs and their mRNA target expressions. To advance our knowledge in this area, single-cell sequencing is necessary in human AD post-mortem samples. Moreover, a recent single-cell sequencing study in the mice hippocampus, although not in an AD-relevant model, suggested that mmu-miR-132 regulates cell-type-specific microglial homeostasis (Walgrave et al., 2023).

Most predominant mature miRNAs, such as hsa-miR-132-3p and hsa-miR-129-5p, are evolutionarily conserved. The sequences of hsa-miR-132-3p and mmu-miR-132-3p are identical (UAACAGUCUACAGCCAUGGUCG), as are the sequences of hsa-miR-129-5p and mmu-miR-129-5p (CUUUUUGCGGUCUGGGCUUGC). However, their predicted targets in humans and mice vary. For hsa-miR-132-3p, there are 673 predicted targets according to miRDB^3^ and 474 according to TargetScan (see text footnote 1), whereas for mmu-miR-132-3p, there are 573 predicted targets according to miRDB and 414 according to TargetScan. For hsa-miR-129-5p, there are 1,417 predicted targets according to miRDB and 732 according to TargetScan, whereas for mmu-miR-129-5p, there are 845 predicted targets according to miRDB and 582 according to TargetScan. In terms of regulation, numerous studies have demonstrated the beneficial impact of miR-132-3p in mice (Smith et al., 2015; Salta et al., 2016; Fatimy et al., 2018), especially in modulating tau pathology, indicating its potential translatability from mouse to human. Additional research is needed to elucidate the impact of miR-129-5p in AD mice models.

In comparison to hsa-miR-132 (Figure 2E; Smith et al., 2015; Salta et al., 2016; Fatimy et al., 2018; Zhang and Bian, 2021), the functional role of hsa-miR-129-5p in AD has received less exploration therefore needs further investigation. However, there is evidence for harmonized expression of hsa-miR-132 and hsa-miR-129 in neurodegenerative disorders. For instance, the downregulation of hsa-miR-132-3p, hsa-miR-129-3p and hsa-miR-129-5p in frozen cerebellar samples of multiple-system atrophy (MSA) patients were reported (Lee et al., 2015). In another cohort a downregulation of hsa-miR-132-3p and hsa-miR-129-5p in formalin-fixed paraffin-embedded samples of cerebellum from MSA patients compared to controls was also observed (Wakabayashi et al., 2016). Furthermore, Parkinson patients treated with dopamine receptor agonists (Pramipexole/ piribedil, L-dopa, amantadine) showed elevated levels of hsa-miR-132 and hsa-miR-129 in the peripheral blood lymphocytes (Alieva et al., 2015). Additionally, in mature primary rat hippocampal neurons, a treatment with GABA-A receptor blocker picrotoxin (PTX) for 48 h resulted in synaptic downscaling and miR-132-3p, miR-132-5p and miR-129-5p were upregulated (Rajman et al., 2017). Intriguingly, in mouse hippocampal neurons both mmu-miR-132 and mmu-129-5p showed neuroprotective mechanisms against Aβ and glutamate toxicity (Fatimy et al., 2018). Untangling in depth mechanisms about the interplay between these two miRNAs in terms of their biogenesis is warranted.

Collectively, these findings significantly enhance our understanding of the dysregulation of specific miRNAs in Alzheimer’s disease. Further investigation into cell-specific miRNA-mRNA target relationships and the associated regulatory pathways, as well as exploring the utility of miRNA expression changes as biomarkers throughout the disease’s progression, may pave the way for potential therapeutic interventions.

## Author contributions

SN: Conceptualization, Funding acquisition, Visualization, Writing – original draft, Writing – review & editing. CQ-S: Conceptualization, Visualization, Writing – original draft, Writing – review & editing. KA: Writing – review & editing. LL: Writing – review & editing. ED: Writing – review & editing. A-CK: Writing – review & editing. EA: Writing – review & editing. J-PB: Writing – review & editing. KL: Conceptualization, Funding acquisition, Supervision, Visualization, Writing – original draft, Writing – review & editing.

## Footnotes

1
www.targetscan.org

2
https://www.synapse.org/#!Synapse:syn3800853

3
www.mirdb.org
